# School-Based Participatory Research with Autistic Children and Young People: A Systematic Review of Approaches, Methods, and Outcomes

**DOI:** 10.1177/13623613261460932

**Published:** 2026-07-21

**Authors:** Chloe Johnson, Clare Wood, David Daley, Lai-Sang Iao

**Affiliations:** 1 Nottingham Trent University, UK

**Keywords:** autism, participatory research, participatory approaches, participatory methods, school, review, reduced inequalities

## Abstract

Participatory research offers a promising means to understand how autistic children and young people (CYP) can be supported and to co-create services or resources that meet their needs. While widely valued, evidence on the most effective ways to meaningfully engage autistic CYP, and on whether and how findings reach educators, is limited. This systematic review narratively synthesises studies reporting the use of school-based participatory research with autistic CYP and reports the approaches, methods, and outcomes of such projects. Initial searches across five databases yielded 1,515 results. After removal of duplicates, 493 studies were screened by abstract and title, and full-text screening was conducted on studies (*n* = 48) that appeared to meet the inclusion criteria. Studies that met pre-identified eligibility criteria (*n* = 22), and studies identified through backward citation (*n* = 2), were included. Creative idea generation, tangible methods, and visual methods are particularly effective for engaging this demographic. However, it was difficult to determine broader implications for schools, as few researchers reported sharing findings with educators. Findings highlight the potential for participatory research, if carefully designed and consistently shared with educators and decision-makers, to contribute to more inclusive schools responsive to the needs and strengths of autistic CYP.

**Lay Abstract:**

Participatory research means working *with* communities rather than doing research *on* them. In the context of autism, this approach involves collaborating directly with autistic children and young people to understand how schools can better support them and to co-create services or resources that meet their needs. While participatory approaches are widely valued, there is limited evidence on the most effective ways to meaningfully involve autistic children and young people in research. In this review, we examined how participatory research has been carried out with autistic children and young people in school settings. We searched five electronic databases for studies published between 2014 and 2024. From these searches, we found 1,515 studies. We then removed studies that were the same, which left us with 493. We then checked the abstract and titles of these 493 studies to see if we should include them in our review. We then read full texts for 48 studies and included 22. By checking the reference lists of the included studies, we identified two additional studies, bringing the total to 24. We found that different ways of involving autistic children and young people in research can work in school settings. Creative idea generation, tangible methods, and visual methods were particularly effective for helping them take part. However, many studies did not report whether findings were shared back with schools, making it difficult to assess broader impacts. Overall, our review highlights the potential for participatory research, when carefully planned and transparently shared with stakeholders, to support schools in becoming more inclusive and better meeting the needs and strengths of autistic students.

## Introduction

Autism has increasingly been recognised as a complex and multifaceted aspect of human diversity. Yet, the ways in which autism is represented have varied across time and context. Traditionally, autism has been understood through a personal tragedy narrative (Singer, 2017), whereby greater emphasis is placed on the autistic person’s challenges, rather than their strengths. This deficit-framing, which rests on the assumption that autistic traits need to be fixed (Monk et al., 2022), has resulted in dominant research paradigms marginalising autistic Children and Young People (hereafter, CYP), where they are often excluded due to their perceived vulnerability^1^ (Cascio et al., 2020; Lewis-Dagnell et al., 2024). Critical Autism Studies foreground issues of epistemic justice, emphasising how different forms of knowledge about autism are produced, legitimised, and contested within unequal power relations (O’Dell et al., 2016; Waltz, 2014). Central to this is the recognition of autistic people as experts in their own lives, alongside “interactional expertise” between researchers and the autistic community (Milton, 2014, p. 794). This involves recognising CYP as important social actors (Bertrand et al., 2020): active participants, rather than providers of information (Ashby, 2011; Montreuil et al., 2021; Sevón et al., 2023).

In recent years, there has been growing interest in participatory research; an “umbrella term” used to describe research approaches that engage those with lived experience (Vaughn & Jacquez, 2020, p. 1). These approaches, including participatory design, participatory action research (PAR), and related terminologies, are underpinned by a commitment to collaborating *with* communities rather than conducting research *on* them (Vaughn & Jacquez, 2020). Within autism research, however, meaningful engagement remains challenging. Communication differences, sensory needs, and the inaccessibility of many traditional research methods can limit the extent to which autistic CYP are able to participate (Lewis-Dagnell et al., 2024; Sharma, 2021). As “creating space” for CYP to meaningfully partake in research is essential to their “empowerment” (Pincock & Jones, 2020), researchers must recognise and accept diverse modes of autistic expression and choose methods that support their participation (Lewis-Dagnell et al., 2024). While choosing an appropriate approach and method is a crucial step to avoid tokenism and facilitate active participation, this decision is difficult to make without clear guidance.

Schools are central to the daily lives and developmental trajectories of autistic CYP, making them a vital context in which to understand their experiences and support needs. Conducting participatory research within school settings enables researchers to engage autistic CYP directly in the environments where learning, social interactions, and support provision occur, generating insights that are immediately relevant to educational practice. Despite this, school-based participatory research has predominantly focused on non-autistic CYP (Montreuil et al., 2021; Sevón et al., 2023) or autistic CYP attending mainstream schools only (Newson et al., 2024). While Newson et al.’s (2024) scoping review offers valuable insight into methods, strengths, and barriers associated with school-based participatory research involving mainstream autistic CYP, further work is needed to systematically examine how such research is enacted across diverse educational contexts and to illuminate the benefits such research may yield both for autistic CYP and the wider school communities. As CYP are recognised as important social actors with the potential to contribute to transformative systemic change (Bertrand et al., 2020), it is essential that their views are not only sought but also listened to and acted upon (Cashmore, 2011). Where research findings are not shared with educators, there is a “significant risk” of co-option (Cornish et al., 2023, p. 10), whereby the voices of CYP are included tokenistically, without influencing decision-making (Tisdall, 2015). To mitigate this, researchers are urged to share findings with the communities involved (Fletcher-Watson et al., 2019). Nevertheless, it remains unclear how, or to what extent, findings from school-based participatory research with autistic CYP are disseminated^2^ to educators. As traditional research outputs are typically peer-reviewed articles, often behind paywalls and inaccessible to stakeholders without university affiliation (Brown, 2024), it is essential to understand how findings are shared with educators and whether any gaps in dissemination are accounted for. Such clarity is crucial to ensure that participatory research transcends academic discourse and individual benefits, driving meaningful change within school communities.

Taken together, these gaps underscore the need for a systematic understanding of the approaches, methods, and outcomes reported in school-based participatory research with autistic CYP across diverse educational contexts. Therefore, the current review systematically summarises extant literature to answer three research questions: (1) What participatory approaches have been used with autistic CYP? (2) What methods enable autistic CYP to be involved in participatory research? and (3) What are the reported outcomes of participatory research projects for autistic CYP and the wider school community? Given anticipated heterogeneity between studies, a summary of our findings is presented narratively under the research questions. Through this, we hope to shed more light on the way participatory research has been used with autistic CYP in school-based settings, and in turn, identify ways in which future research could be enhanced.

## Method

The review followed the Preferred Reporting Items for Systematic Reviews and Meta-Analysis (PRISMA) guidelines (Moher et al., 2015). A study protocol was registered on the Open Science Framework (osf.io/jnp2d) on February 16, 2025, prior to data extraction.

### Search Strategy

PROSPERO and the Open Science Framework were searched to ensure that no systematic review on this topic was underway. Five databases (SCOPUS; PUBMED; Web of Science; PsychInfo; Eric) were searched to assess if key search terms generated appropriate results published between 2014 and 2024 in the databases. The year 2014 was chosen as the starting point for the literature search because it marks both the re-definition of autism in the *Diagnostic and Statistical Manual of Mental Disorders* (5th ed.; DSM-V) and a period of growing recognition of the importance of participatory approaches in autism research, particularly in response to critiques of traditional, researcher-led methods that often excluded autistic voices (Greenstein, 2014; Milton et al., 2025; Pellicano et al., 2014). After initial searches, 10 search terms, which included a combination of keywords with Boolean logic and truncation, were revised through team discussion (see Supplemental Appendix 1). Formal searches across the same five databases were conducted on January 1, 2025, and the results were filtered by abstract, title, and keywords.

### Eligibility Criteria

The inclusion and exclusion criteria (see Table 1), decided a priori, were carefully designed to capture a breadth of studies reporting use of participatory research with autistic CYP (aged 4–18) in school settings. Studies that self-identified as “participatory,” and/or studies that aligned with the core principles of participatory approaches, that is, working collaboratively with autistic CYP rather than conducting research on them, were included. To ensure relevancy, age parameters were implemented to account for the earliest age CYP start school and the highest compulsory leaving age (Right to Education Initiative, 2004), and studies were restricted to those conducted in school settings. While team language proficiency meant that it was necessary to exclude studies published in non-English languages, no restrictions were placed on the country of publication.

**Table 1.** Inclusion and Exclusion Criteria.

Inclusion criteria	Exclusion criteria
Written in the English language	Not written in the English language
Peer-reviewed article	Conference proceedings; theses and dissertations; unpublished reports; lecture notes; study protocols; reviews; books
Methods and outcomes of the research are clearly defined	No clear outline of the methods used and outcomes of the project.
Study reported using participatory research with autistic CYP (4–18) in a school setting	Study participants outside of our age range. Studies that do not report using participatory research. Studies conducted in settings outside of school.

### Study Selection

Search results were imported into Zotero, and duplicates were removed using ASySD (Hair et al., 2023). Unique citations were then re-imported into Zotero, and C.J. screened studies by abstract/title using the pre-identified eligibility criteria. For inter-rater reliability, L.-S.I. checked a random sample of 20%. Following random sample checking, C.J. and L.-S.I. compared results, with a >98% agreement score. C.J. and L.-S.I. then independently conducted full-text screening on papers that remained after abstract/title screening. A couple of disagreements arose; these were discussed between C.J. and L.-S.I. and sent to a third reviewer (D.D.) for arbitration. The arbitration resulted in one being excluded and one being retained. Studies that met the inclusion criteria outlined in Table 1 were included in the review.

### Data Extraction

Data from studies that met the pre-identified inclusion criteria (*n* = 22), and studies identified through backward citation (*n* = 2), were extracted. Extracted data were organised into a bespoke table, using word processing software, under the following headings: (1) author(s) and year; (2) country; (3) sample size and demographics; (4) quality rating; (5) approaches and method; and (6) outcome.

### Assessment of Study Quality

Studies included in this review were evaluated for methodological quality using the Mixed-Methods Appraisal Tool (MMAT), which allowed us to provide assessments on qualitative, quantitative, and mixed-methods studies (Hong et al., 2018). The five MMAT quality criteria differ based on study design. Each criterion is rated as “Yes” (meets the criterion), “No” (does not meet the criterion), or “Can’t tell” (insufficient information reported). As Hong et al. (2018) caution against percentage scoring, how the studies met the criteria is presented in Supplemental Appendix 4. All studies satisfied the initial two screening questions. Qualitative studies often did not clearly demonstrate that their interpretations of results were sufficiently substantiated by the data. Mixed-methods studies tended to be unclear on how the qualitative and quantitative components were integrated, with only two studies explicitly addressing how these complimented or diverged from one another. Given that all studies met almost all criteria, no studies were excluded following the quality assessment.

### Positionality Statement

This review was carried out by a collaborative team that included autistic and non-autistic researchers, all contributing lived experience and academic expertise in autism. The first author is an autistic doctoral student with prior experience as a teacher of English at the secondary education level.

### Data Availability Statement

All data are available in the Supplemental Appendix or from the corresponding author upon reasonable request.

## Results

A total of 1,515 studies were identified with the above search strategy. In all, 493 studies were screened for inclusion after removal of duplicates. In total, 27 studies were excluded following full-text screening, two studies were located through backward citation, and 24 studies were included in the review (see Figure 1). A full study table is presented in Supplemental Appendix 3.

**Figure 1.**
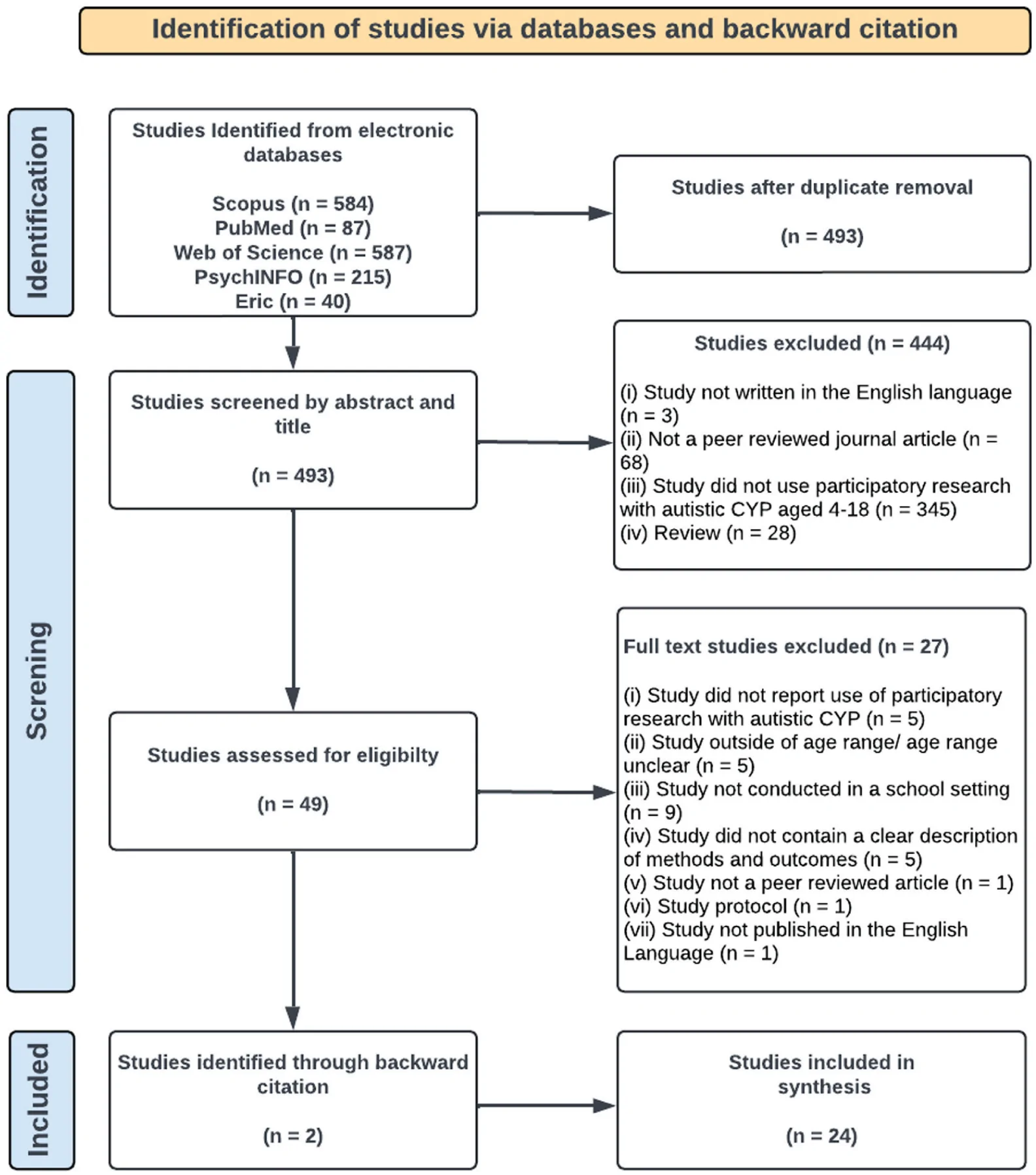
PRISMA flowchart showing study identification process (Moher et al., 2015).

### Characteristics of Studies

The majority were published in the United Kingdom (*n* = 17). Other studies were conducted in Australia (*n* = 2), Turkey (*n* = 1), Finland (*n* = 1), Ireland (*n* = 1), Chile (*n* = 1), and France (*n* = 1). In total, 75% of these were qualitative, 20.8% were mixed-methods, and one were quantitative. Across the studies, participants (total *n* *=* 234) were from mainstream (*n* = 12) and special educational provisions (*n* = 11). One study (Goodall, 2018) included autistic CYP from mainstream and home-school provisions, and another study (Suero Montero et al., 2023) did not state where participants were drawn from. Of those who gender reported, 17.4% were female and 82.6% were male. Where co-occurring conditions were reported, these included intellectual disability (ID), physical disability, epilepsy, hyperactivity, hyposensitivity, and Attention-Deficit Hyperactivity Disorder (ADHD). Eleven studies adopted a medicalised view of autism, with only three situated within the neurodiversity model. Ten studies used identity-first language, although their underlying philosophical stance was often unclear.

### What Participatory Approaches Have Been Used with Autistic CYP?

Included studies were mapped against the non-exhaustive list of participatory research approaches outlined by Vaughn and Jacquez (2020). The primary used approach was participatory design (*n* = 12). Across these studies, participatory design was characterised by an iterative and collaborative process in which autistic CYP were actively engaged in structured design activities that supported idea generation, reflection, and refinement across multiple sessions. In line with participatory design principles, these studies emphasised shared decision-making, responsiveness to participant feedback, and adaptation of activities to meet participants’ communication preferences and sensory needs. While the extent of involvement varied, autistic CYP were most commonly engaged during the action stage of the research, with fewer studies involving them in early planning or later stages of evaluation. While similarly iterative and collaborative, action research differs from participatory design in that it focuses on addressing practical concerns through cycles of action and reflection (Vaughn & Jacquez, 2020). One study reported the use of action research (Tyrrell & Woods, 2019), which involved autistic CYP actively organising their exam access arrangements across action and reflection stages, enabling them to have greater say in how they were supported in their school.

Eleven studies did not map onto Vaughn and Jacquez’s (2020) non-exhaustive list, yet seemed to share characteristics with PAR, as they appeared focused on centralising lived experience (Fernández, 2022, p. 37) to reduce educational inequalities (Dedding et al., 2021) through implicit cycles of planning, action, and reflection. To avoid the risk of misinterpretation (Clearys et al., 2016), we chose not to ascribe a specific framework to these studies. Instead, we focus on the shared characteristics and implicit stages (see Table 2).

**Table 2.** Characteristics of Studies That Did Not Map onto Vaughn and Jacquez’s Non-Exhaustive List (Adapted from Freire et al., 2022).

Reference	Terminology used	Implicit stages used in the study	Stage(s) in which autistic CYP were involved
Danker et al. (2016)	Participatory techniques	Planning, action, reflection	Action, reflection
Danker et al. (2019)	Participatory research method	Planning, action, reflection	Action, reflection
Fleming et al. (2023)	Participatory research	Planning, action, reflection	All
Glass et al. (2024)	Participatory research/co-construction	Planning, action, reflection	Action, reflection
Goodall (2018)	Participatory approach	Planning, action, reflection	Action, reflection
Hoy et al. (2018)	Co-construction	Planning, action, reflection	Action, reflection
Istuany and Wood (2020)	Participatory methods/approaches	Planning, action, reflection	Action, reflection
Molloy and Farrell (2024)	Participatory research	Planning, action, reflection	Action
Newbutt and Bradley (2022)	Participatory research/co-participation	Planning, action, reflection	Action, reflection
Parsons et al. (2021)	Co-construction	Planning, action, reflection	Action
Suero Montero et al. (2023)	Participatory research/approach/strategies	Planning, action, reflection	Action, reflection

While these studies were united in their commitment to better understanding and/or improving autistic CYP’s educational experiences, the stages in which CYP were involved differed. Two studies only involved autistic CYP in the action stage, which was broadly synonymous with data collection. Where autistic CYP were involved in action and reflection stages, this generally involved them completing an activity before reflecting on their experiences. Only one study (Fleming et al., 2023) involved autistic CYP in planning the study design, yet autistic adults were occasionally consulted (Suero Montero et al., 2023). Two studies (Danker et al., 2019; Istuany & Wood, 2020) stated that they used participatory methods but did not appear to self-identify as participatory. Nevertheless, their focus on capturing the lived experiences of autistic CYP, alongside facilitating meaningful engagement, aligns with core participatory research principles.

### What Methods Enable Autistic CYP to Be Involved in Participatory Research?

To enable greater comparability to non-autistic CYP where possible, we classified methods under the same categories as Sevón et al. (2023). Among the included studies, methods included traditional methods such as observation methods and language-based methods and creative methods such as creative idea generation, technology-based methods, visual methods, and autistic CYP as co-researchers. Studies reporting the sole use of traditional methods were uncommon (see Table 3). Two studies (Istuany & Wood, 2020; Newbutt et al., 2023) reported the use of focus groups. While focus groups could be considered as a distinct methodological category, the above categories were sufficient to classify these studies.

**Table 3.** Method Distribution (Marked ✓ to Indicate Where Methods Were Used).

Reference	Traditional methods		Creative methods			
	Observation	Language-based methods	Creative idea generation	Technology-based methods	Visual Methods	Autistic CYP as co-researchers
Benton and Johnson (2014)		✓	✓			
Bossavit & Parsons (2017)		✓	✓			
Bossavit & Parsons (2018)	✓	✓				
Constantin et al. (2017)		✓	✓			
Danker et al. (2016)		✓			✓	
Danker et al. (2019)		✓			✓	
Fage et al. (2019)	✓					
Fleming et al. (2023)					✓	✓
Glass et al. (2024)					✓	
Goodall (2018)		✓	✓			
Hoy et al. (2018)		✓			✓	
Hummerstone & Parsons (2022)		✓	✓			
Istuany & Wood (2020)		✓	✓			
Lemaignan et al. (2024)	✓	✓		✓		
McAllister & Sloan (2016)		✓	✓			
Merter & Hasirci (2018)	✓					
Molloy & Farrell (2024)	✓					✓
Newbutt & Bradley (2022)		✓				
Newbutt et al. (2023)	✓	✓				
Parsons (2015)	✓					
Parsons et al. (2021)					✓	
Suero Montero et al. (2023)		✓		✓		
Tyrrell & Woods (2019)		✓	✓			
Ward et al. (2022)		✓	✓		✓	

### Observation Methods

While some studies supplemented observation methods with language-based methods, others relied solely on observation, drawing on accounts of autistic CYP provided by adults in their care (Ashby, 2011). These accounts were captured through video recordings, direct observations, and interviews or questionnaires with educators. Although participatory research aims to move participants from being observed phenomena to active contributors, challenges remain when involving non-speaking^3^ autistic CYP. For instance, where CYP were non-speaking (Merter & Hasirci, 2018), educator and researcher observations allowed for data to be collected and interventions to be shaped in line with perceived needs. Nevertheless, Parsons et al. (2021) demonstrated that the inclusion of non-speaking autistic CYP is possible through the use of digital stories.

### Technology-Based Methods

Two studies reported the use of technology-based methods. This included eye-tracking to measure participant gaze during social interactions, video recordings of participant interactions (Suero Montero et al., 2023), and automated data collection to record instances of autistic CYP interacting with a social robot (Lemaignan et al., 2024). However, automated data collection proved unreliable and was replaced with researcher observations (Lemaignan et al., 2024).

### Language-Based Methods

Seven studies reported using questionnaires. Standardised questionnaires were difficult for autistic CYP to comprehend (Newbutt & Bradley, 2022), and open questions were not completed (Suero Montero et al., 2023). Nevertheless, autistic CYP were able to engage with child-friendly Likert-type scales (Benton & Johnson, 2014; Lemaignan et al., 2024). While researchers were on hand to clarify questions (Bossavit & Parsons, 2018), no difficulties were reported when educators, parents, or autistic people (Istuany & Wood, 2020; Lemaignan et al., 2024; Newbutt & Bradley, 2022) were involved in the design or piloting of questionnaires.

More commonly, in-person questioning was used; these were documented through verbal questioning, discussion, and interviews. Verbal questioning was used to guide idea formulation and reflection (Bossavit & Parsons, 2017; McAllister & Sloan, 2016). Where questions were abstract, participation was supported through visual prompts, which involved researchers showing pictures (Tyrrell & Woods, 2019). At other times, autistic CYP were required to answer written questions on worksheets (Constantin et al., 2017) and complete mind-maps^4^ (Benton & Johnson, 2014; Newbutt et al., 2023), which often required the support of educators to clarify questions and guide responses.

In the two studies that used group discussion, autistic CYP were able to (1) express their views towards a social robot (Newbutt et al., 2023) and (2) design games (Ward et al., 2022). Participation was supported through tangible and visual prompts, including inviting autistic CYP to interact with a social robot (Newbutt et al., 2023). When autistic CYP were engaged in individual discussions with the researcher, they provided annotations on a “motivational mixer” worksheet (Hummerstone & Parsons, 2022). Similarly, in studies reporting the use of interview, participation was supported through visual prompts, including photographs, pictures, or videos (Danker et al., 2016, 2019; Hoy et al., 2018; Tyrrell & Woods, 2019); ranking and drawing activities (Goodall, 2018); and co-producing questions with autistic CYP (Ward et al., 2022). While no specific support was reported by Istuany and Wood (2020), it is possible that autistic CYP were able to draw upon previous focus group sessions, which incorporated coloured pens and photographs, to support their responses.

### Creative Idea Generation

Eight studies used creative idea generation: these methods included artefact creation (Ward et al., 2022), prototype development (Benton & Johnson, 2014; Bossavit & Parsons, 2017; Constantin et al., 2017), and activities using tangible materials (Constantin et al., 2017; Goodall, 2018; Istuany & Wood, 2020; Tyrrell & Woods, 2019). While prototype development was limited to studies incorporating a participatory design approach, other methods were used across a range of participatory approaches, suggesting versatility. Where difficulties arose, these appeared to stem from researchers being less experienced in engaging CYP (Bossavit & Parsons, 2017).

Drawing was used in three studies and enabled autistic CYP to share their ideas regarding their ideal school (Goodall, 2018; Istuany & Wood, 2020) and preferred exam access arrangements (Tyrrell & Woods, 2019). Drawing was also incorporated into prototypes (Constantin et al., 2017), and, although initially selected as a method by co-researchers, resulted in irrelevant drawings and was subsequently abandoned (Fleming et al., 2023). While participants generally engaged in drawing activities unsupported, difficulties arose when one secondary-aged student did not enjoy drawing and was reluctant to do it (Tyrrell & Woods, 2019). No reluctance was noted for sorting activities (*n* = 3), including diamond rankings (Goodall, 2018) and card sorts (Constantin et al., 2017; Tyrrell & Woods, 2019); autistic CYP could complete these without adult support. However, input from educators was sought to ensure task suitability (Constantin et al., 2017).

### Autistic CYP as Co-Researchers

Two studies (Fleming et al., 2023; Molloy & Farrell, 2024) reported that autistic CYP were co-researchers; however, the level of involvement differed. While autistic CYP in Fleming et al. (2023) were involved in the planning and data analysis stages, their involvement in Molloy and Farrell (2024) appears to have been limited to goal setting, guided reflection, and collaboration with teachers to address barriers to their behavioural goals.

### Visual Methods

Where autistic CYP were invited to act as co-researchers (Fleming et al., 2023), visual methods, specifically the use of images, were selected after the drawing task elicited irrelevant data. In addition, three studies used photovoice, which served as an effective medium for eliciting the voices of autistic CYP and allowed them to confidently share their views regarding wellbeing (Danker et al., 2016, 2019) and school transitions (Hoy et al., 2018). Essentially, the photovoice studies followed the same format, with autistic CYP either taking photographs around the school site or providing images; these were then analysed thematically. While the majority of these studies did not include autistic CYP in the data analysis stage, the use of a child-friendly approach to data analysis in Fleming et al. (2023) allowed them to participate in all stages. This approach involved autistic CYP collating pictures and memes on Padlet, which were then screen-captured and organised into themes.

More commonly, studies (Danker et al., 2016, 2019; Hoy et al., 2018) used participant-supplied photographs during interviews, providing autistic CYP an opportunity to comment on the photographs they had taken, which encouraged self-awareness and reflection on their school experiences. Photo-elicitation interviews promoted ease by removing the barrier of making eye contact during the interview and empowering autistic CYP to take ownership of their photographs (Danker et al., 2016). However, some participants provided brief responses during the interview, which meant that the researchers had to use “probing questions” (Danker et al., 2016, p. 22). Other challenges included students forgetting to take photographs and educators suggesting and restricting the photographs taken (Danker et al., 2016). These issues were avoided when students took photographs in the presence of a researcher (Hoy et al., 2018).

Three studies (Glass et al., 2024; Parsons et al., 2021; Ward et al., 2022) reported use of digital stories to document the experiences of autistic CYP. For younger, non-speaking participants, footage was captured using wearcams attached to students’ shirts, which was then coded and edited by the research team. In contrast, older participants (Glass et al., 2024; Ward et al., 2022) were involved in the development and editing process.

### What Are the Reported Outcomes of Participatory Research Projects for Autistic CYP and the Wider School Community?

Participatory design allowed autistic CYP to design or evaluate games (Bossavit & Parsons, 2017, 2018; Parsons, 2015; Ward et al., 2022), interventions (Merter & Hasirci, 2018), reward systems (Constantin et al., 2017), school layouts (McAllister & Sloan, 2016), apps (Fage et al., 2019), information sheets (Hummerstone & Parsons, 2022), and a social robot (Lemaignan et al., 2024; Newbutt et al., 2023). Autistic CYP were also able to capture their experiences of participatory design processes (Ward et al., 2022). Participatory design was, in some cases, also associated with skill development. For instance, autistic CYP in Bossavit and Parsons’ (2017) study designed a game which allowed them to meet “curriculum requirements” for “programming” (p. 5).

Other studies, which did not employ participatory design approaches, were generally focused on understanding autistic CYP’s experiences, needs, and preferences. Six studies supported a more holistic understanding of autistic CYP, including wellbeing (Danker et al., 2019), educational experiences (Glass et al., 2024), social interactions (Lemaignan et al., 2024; Newbutt et al., 2023; Suero Montero et al., 2023), and views towards virtual reality (Newbutt & Bradley, 2022). Involvement led autistic CYP in one study (Glass et al., 2024) to become better connected to the community, with one student gaining future work experience. Three of these studies (Glass et al., 2024; Newbutt & Bradley, 2022; Suero Montero et al., 2023) commented on the empowerment granted by participatory research.

Other studies helped autistic CYP to express their educational preferences (Constantin et al., 2017; Fleming et al., 2023; Goodall, 2018; Hoy et al., 2018; Hummerstone & Parsons, 2022; Istuany & Wood, 2020; McAllister & Sloan, 2016) and become more involved in decisions regarding exam access arrangements (Tyrrell & Woods, 2019). Barriers within school were also emphasised by autistic CYP in six studies (Danker et al., 2016; Goodall, 2018; Hoy et al., 2018; Hummerstone & Parsons, 2022; Istuany & Wood, 2020; McAllister & Sloan, 2016), who expressed their distaste for noise, both within the classroom and on the corridors. Potential solutions were offered by autistic CYP, including the need for quiet spaces (Istuany & Wood, 2020) and appropriate locations for a support hub (McAllister & Sloan, 2016).

### Dissemination and Real-World Impact

Sharing findings with educators is a crucial step to ensure participatory research is meaningful to the communities involved. However, only four studies explicitly reported sharing findings with educators, and no authors accounted for not disseminating. At times, dissemination activities were ambiguously reported. For instance, Danker et al. (2019) reported that participants were assured their findings would be shared with schools via a report, yet did not specify whether findings were ultimately disseminated. Similarly, when educators were also participants, or involved in the conceptualisation of the research, it was sometimes unclear whether findings were shared beyond those directly involved (Hoy et al., 2018; Lemaignan et al., 2024; Newbutt et al., 2023; Tyrrell & Woods, 2019).

Where authors explicitly reported sharing findings, dissemination activities included sharing information sheets and eliciting feedback from educators (Hummerstone & Parsons, 2022), presentations to educators, lay summaries published on the School Trust’s website (Newbutt & Bradley, 2022), and sharing digital stories with educators (Glass et al., 2024; Parsons et al., 2021). In these cases, outcomes were mostly positive, including the incorporation of findings into the schools’ strategic planning (Newbutt & Bradley, 2022) and a greater understanding of ways in which autistic CYP can be supported (Glass et al., 2024; Hummerstone & Parsons, 2022; Parsons et al., 2021). However, educators noted that time and resource constraints could make it difficult to implement findings (Hummerstone & Parsons, 2022). It was also unclear whether findings reached educators beyond the staff who provided feedback (Hummerstone & Parsons, 2022).

## Discussion

This systematic review synthesised the approaches, methods, and outcomes of 24 studies reporting use of school-based participatory research with autistic CYP. Three of these studies (Danker et al., 2016, 2019; McAllister & Sloan, 2016) were also included in Newson et al. (2024). Their findings regarding gender biases, the influence of teachers on data collection, and the benefits of participatory research for individual autistic CYP are broadly consistent with ours. However, our review advances the field in several ways. First, we found that various participatory approaches have been used, yet some studies did not ascribe a specific framework, which created ambiguity. We also found that some methods, particularly language-based methods, were often inaccessible without adult support, yet creative idea generation, visual methods, and tangible methods supported autistic CYP to meaningfully engage in research with little to no adult support. However, it was difficult for us to determine outcomes concerning wider school inclusion, as only few studies reported sharing findings with educators. These findings align with principles from Critical Autism Studies, which help explain why inconsistencies in participation remain evident across studies. The structural and interpersonal power imbalances that shape research encounters, including educator influence during data collection and limited autistic involvement in planning or reflection stages, were observed within several studies in this review. From this perspective, future school-based participatory research must do more than adopt inclusive methods. It should actively work to redistribute power and foreground autistic ways of knowing. This includes being explicit about the philosophical approach guiding participatory work, embedding autistic expertise throughout the research process, and avoiding language or practices that implicitly pathologise autism. In the following, we discuss key findings and recommendations in relation to the wider literature and comment on the ways future school-based participatory research could be enhanced.

First, researchers should identify which participatory approach their work best aligns with, rather than relying on umbrella terms (i.e., participatory research, co-construction, and participatory approaches), as each is conceptually distinct (Vaughn & Jacquez, 2020) and blurring boundaries can create ambiguity (Montreuil et al., 2021). Our review found inconsistencies in how studies described involvement of autistic CYP, with some limiting participation to data collection, while others included reflection stages. While data collection is important, PAR must extend beyond this to ensure meaningful change (Fernández, 2022); without reflection, participation risks being merely consultative (Lloyd-Evans et al., 2023; Vaughn & Jacquez, 2020). Although many studies did not explicitly claim to use PAR, adopting a clear framework would provide consistency and set expectations for levels of involvement. In this review, participatory design was most common, often suited to developing tangible outputs such as technology-based tools (Broadley & Dixon, 2022), whereas PAR may be more appropriate for research aiming to improve educational experiences and raise awareness of autistic CYP needs (Fernández, 2022; Jacobs, 2016). Where PAR is adopted, researchers should ensure meaningful engagement of CYP throughout the process, including planning, to reflect CYP preferences (Coyne & Carter, 2024). For further guidance, Vaughn and Jacquez (2020) offer useful direction.

Second, researchers should carefully select methods that enable autistic CYP to become meaningfully involved, such as creative idea generation, visual methods, and tangible methods. Concerning methods, we found, in line with Sevón et al. (2023), that language-based methods were most common, with 68% of studies reporting their use. While studies were mostly sensitive to the needs of autistic CYP, their use of standardised questionnaires, particularly open-ended ones, was still challenging for autistic CYP. This aligns with Stacey and Cage (2023), who noted difficulties due to unclear wording and decision-making demands, which can be effortful for autistic people (Vella et al., 2018). Involving educators, parents, or autistic people in the design or piloting of questionnaires may help reduce these difficulties.

In many studies, autistic CYP engaged with research methods when supported by adults, often educators. While educator participation can be beneficial, it may also introduce potential biases, as students are typically socialised to respect authority (Bertrand & Lozenski, 2023) and may hesitate to share genuine views. This dynamic can create a “halo-effect” (Flick, 2015, p. 137), whereby responses are unconsciously shaped by perceived expectations. Our review noted instances where educators inadvertently influenced data collection processes, such as restricting or suggesting photographs during photovoice (Danker et al., 2016). Nevertheless, educators also fostered positive learning climates (Benton & Johnson, 2014; Bossavit & Parsons, 2017). To ensure that the influence of educators remains supportive, not dominative, researchers should clearly define expectations for educator involvement from the outset.

Notably, methods which played to the strengths of autistic CYP enabled independent participation. Consistent with literature highlighting autistic CYP’s enhanced responsiveness to visual media (Rentschler et al., 2024) and systemising tasks (Baron-Cohen, 2006), effective methods included photographs, drawing, and sorting activities. Yet, as adolescents may feel dissatisfied with drawings (Richards, 2003), one student was reluctant to draw. Researchers should therefore consider age-appropriate options for self-expression. Level of input for digital stories was also affected by age and cognitive capabilities, with secondary-aged mainstream autistic CYP being more actively involved but requiring extended timeframes (Glass et al., 2024). This aligns with Kaptan and Cakir’s (2025) finding that the creation of digital stories took longer for autistic CYP with lower levels of technological proficiency. Given school time pressures (Halliday et al., 2018), researchers should be transparent with educational providers to ensure they understand the expected level of commitment. In contrast, photovoice, being relatively time-efficient (Hoy et al., 2018) and effective for communicating needs (Wang & Burris, 1997), may be preferable when resources are limited. When using photographs and systemisation tasks, autistic CYP were able to articulate their reasoning with minimal adult input, given the increased thinking time involved in such methods. Lewis-Dagnell et al. (2024, p. 8) also reported that such activities reduced pressure from “on the spot” responses, which otherwise place heavy demands on working memory (Barendse et al., 2013). Challenges remained, such as students forgetting to take photographs in photovoice (Danker et al., 2019), which could be mitigated by researcher support (Hoy et al., 2018).

Interestingly, while Sevón et al. (2023) found that 11 studies invited non-autistic CYP as co-researchers, only two studies (Fleming et al., 2023; Molloy & Farrell, 2024) included in our review reported use of this, and the level in which autistic CYP were involved differed. While positive findings from Fleming et al. (2023) suggest that autistic CYP can become involved in the data analysis stage, the value of participatory data analysis remains contentious (Nind, 2011), and it was not possible for us to conclude, from one study, whether involving autistic CYP in the data analysis stage would enhance analytic processes or present methodological challenges. We therefore outline this as a topic of interest for further research.

Third, researchers should commit to sharing findings to maximise the benefits for the wider community, as these efforts can help bridge the disconnect between research and practice (Tripathy et al., 2017). We found that where findings were disseminated to educators, schools often developed a better understanding of how autistic CYP could be supported and implemented changes in practice. Yet, few studies reported sharing findings with educators, leaving the wider school impact unclear. Word limits in academic outputs may partly explain this gap (Dedding et al., 2021; Freire et al., 2022), but greater transparency concerning community outcomes would help illuminate the real-word benefits afforded by participatory research. Freire et al. (2022) proposed developing reporting guidelines for participatory research, which future research could explore.

When disseminating findings to educators, researchers should also consider the practical realities in schools. While participatory research aims to produce findings that are relevant and actionable for communities (Gollust et al., 2025), educators often face intense workloads and “time poverty” (Education Support, 2025, p. 44) and have expressed a preference for concise formats (Barrett & Prendergast, 2025). As time and resource constraints may limit uptake (Barrett & Prendergast, 2025) even when research is deemed relevant (Lysenko et al., 2015), information should be concise and appropriately formatted with practical takeaways (Barrett & Prendergast, 2025; Chen et al., 2010; Henriksen & Mishra, 2019). Alongside the dissemination routes reported in our review, other options could include interactive presentations (McDavitt et al., 2016) or practitioner journals written in accessible styles (Henriksen & Mishra, 2019). Ultimately, dissemination strategies should be co-designed with educators to ensure relevance and usability.

Finally, researchers should carefully consider their language and framing of autism to ensure participatory research is respectful to the autistic CYP involved. While participatory research has been associated with “lower levels of ableist cues” (Botha & Cage, 2022, p. 4), many studies classified autism in terms of impairments and deficits. Unequal power dynamics between children and adults (Bertrand & Lozenski, 2023) may partially explain this, as CYP may have had limited influence on how autism was represented in research outputs. Going forward, we recommend that participatory researchers consult Monk et al. (2022) for guidance on conducting research that avoids framing autism as a pathology.

### Strengths, Limitations, and Future Research

This systematic review of participatory research with school-aged autistic CYP provides insight into approaches, methods, and outcomes, offering guidance for future studies. By searching five databases across education and psychology, we captured interdisciplinary perspectives, although the review has limitations. First, participatory research is an “umbrella term” that encompasses a range of related concepts, including “co-design,” “co-creation,” “co-research,” “co-production,” and “action research.” While we included specific searches for “action research,” our overall search strategy primarily relied on the term “participatory.” As a result, studies employing participatory principles under alternative terminology may not have been captured sufficiently. Future reviews could therefore consider broadening search terms to ensure more comprehensive coverage of the field. Second, our review was limited to peer-reviewed English publications. Future reviews could expand to non-education contexts, non-English studies, and differentiated approaches to data analysis, which remain a “missing dimension” in participatory research (Nind, 2011, p. 352). Sample representation also requires attention. While autistic females are often underdiagnosed, comprising about 25% of the autistic populations (Loomes et al., 2017), our review found that 17.4% were female. In addition, studies involving autistic CYP in school-based participatory research remain low, indicating a need for greater inclusion of autistic CYP in future research. Moreover, as some studies included participants from primary and secondary schools, it was not possible for us to determine which methods are most effective for specific ages. Future research could evaluate methods across different age groups, as needs and preferences may differ. Given evidence that autistic CYP can engage meaningfully when supported (Cascio et al., 2020), researchers should prioritise inclusive practices. Creative idea generation, tangible methods, and visual methods, such as photovoice, drawing, and sorting, are effective in facilitating authentic expression from autistic CYP with minimal adult input.

## Conclusion

Our review did not seek a panacea for inclusive participatory research but to support researchers in selecting approaches and methods suited to the autistic CYP they work with. Going forward, participatory researchers are encouraged to align their work with a defined approach to enhance consistency. In school contexts, adult involvement to ensure accessibility is beneficial, although educators’ role in supporting autistic CYP engagement must be clearly defined to avoid bias. Crucially, autistic CYP voices should reach decision-makers through agreed dissemination routes to help narrow the disconnect between research and practice. Above all, future work must embed autistic expertise throughout the research process and avoid language or practices that implicitly pathologise autism. Findings from our review suggest that participatory research, when carefully designed and transparently reported, has the potential to create more inclusive schools attuned to the needs and strengths of autistic CYP.

## Supplemental Material

sj-docx-1-aut-10.1177_13623613261460932 – Supplemental material for School-Based Participatory Research with Autistic Children and Young People Supplemental material, sj-docx-1-aut-10.1177_13623613261460932 for School-Based Participatory Research with Autistic Children and Young People by Chloe Johnson, Clare Wood, David Daley and Lai-Sang Iao in Autism

## Notes

1. For the purpose of this article, the term vulnerable is used to describe “categories of people [who] are presumed to be more likely than others to be misled, mistreated, or otherwise taken advantage of as participants in research” (Levine et al., 2004, p. 44).

2. Our understanding of dissemination is informed by the definition provided in Ross-Hellauer et al. (2020): “communicating and interacting with wider audiences in ways that will facilitate research uptake and understanding” (p. 2). For the purpose of this article, dissemination refers to researchers’ activities, beyond academic publishing, that ensure findings reach educators.

3. Following recommendations from Bottema-Beutel et al. (2025), we use the term non-speaking rather than non-verbal throughout. For further details, we urge readers to consult this work.

4. Some studies used the term “brainstorm” rather than “mind-map,” yet as these terms are interchangeable, we grouped these under “mind-map” for ease.
