# Supplementary material for: School-Based Participatory Research with Autistic Children and Young People: A Systematic Review of Approaches, Methods, and Outcomes
Source: Autism. 2026 Jul 21;30(11):2725–38. doi: 10.1177/13623613261460932 (PMC13687619; doi:10.1177/13623613261460932)
Supplement: Supplementary file 1 [file sj-docx-1-aut-10.1177_13623613261460932.docx]

**Appendices**

**APPENDIX 1.** Search strategy including search terms, databases searched, and results yielded.

|  | Searches | SCOPUS | | PUBMED | Web of Science | PsycINFO | Eric |
| --- | --- | --- | --- | --- | --- | --- | --- |
| 1 | (Participatory Research) AND (autis* OR asd OR ASC) AND (school) | 78 | 17 | | 66 | 35 | 3 |
| 2 | (Participatory Research) AND (autis* OR asd OR ASC) AND (child* or adolescen*) AND (school) | 59 | 11 | | 59 | 24 | 0 |
| 3 | (participatory research) AND (special AND educational AND need) AND (school) | 48 | 1 | | 41 | 20 | 31 |
| 4 | (participatory) AND (autis* OR asd OR asc ) AND ( child* OR adolescen* ) AND ( school ) | 75 | 21 | | 77 | 37 | 2 |
| 5 | (participatory research) AND ( neurodiv* ) AND ( school ) | 9 | 1 | | 8 | 1 | 0 |
| 6 | "Participatory Action Research" AND ( autis* OR asc OR asd ) AND (child* or adolescen*) | 6 | 4 | | 18 | 12 | 0 |
| 7 | "Participatory Design" AND ( autis* OR asc OR asd ) AND ( child* OR adolescen* ) | 101 | 13 | | 99 | 20 | 2 |
| 8 | "Action research" AND ( autis* OR asc OR asd ) AND ( child* OR adolescen* ) | 43 | 4 | | 49 | 21 | 0 |
| 9 | "community-based participatory research" AND ( autis* OR asc OR asd ) AND ( child* OR adolescen* ) | 26 | 11 | | 24 | 10 | 1 |
| 10 | (participatory approaches) AND ( autis* OR asc OR asd ) AND ( child* OR adolescen* ) | 139 | 4 | | 146 | 35 | 1 |
| Total | | 584 | 87 | | 587 | 215 | 40 |

| **APPENDIX 2.** Excluded articles and reasons for exclusion | |  |
| --- | --- | --- |
| **Reference** | **Reason for exclusion** | |
| Adderley et al. (2014) | While the study used participatory research to explore CYP perceptions toward inclusion, the authors do not refer to autistic CYP. | |
| Ashburner et al. (2023) | This study was not conducted in a school setting. | |
| Barakova et al. (2015) | This study was conducted in a clinical setting. | |
| Clément et al. (2022) | This study was not conducted in a school setting. | |
| Ferreira & Muniz (2024) | Not participatory research. | |
| Frauenberger et al. (2018) | No clear description of the methods and outcomes. | |
| Ruth (2020) | Dissertation | |
| Guldberg et al. (2024) | Ages of CYP included in the research not provided. Paper only states age range of CYP supported in the schools (3-19), which extends beyond our age range. | |
| Hill et al. (2016) | CYP aged 13-19; study did not detail how specific ages were involved in the research. Additionally, the study looked at a variety of SEND needs and did not explain the specific outcomes for autistic CYP. | |
| Kerr, et al. (2022) | No clear description of the methods used to engage autistic CYP, and no comment on how these were received. | |
| Lam et al. (2020) | Study was designed to support autistic youth aged 19-25 in post-secondary transitions. | |
| Lewis-Dagnell et al. (2024) | Study conducted in a residential setting for autistic adults. | |
| Maravé et al. (2021) | Study published in Spanish and not available in the English language. | |
| Mogensen & Mason (2015) | This study was not conducted in a school setting. | |
| Neilson & Bond (2024) | Study conducted in an alternative setting for school non-attenders. | |
| O’Hagan & Byrne (2023) | This study was not conducted in a school setting. | |
| Parsons et al. (2014) | No clear description of the methods used to engage CYP in participatory design. | |
| Parsons et al. (2015) | Study with educators | |
| Parsons et al. (2023) | Study protocol | |
| Pui (2024) | Study focuses on SEND CYP generally, and not specifically on autistic CYP. | |
| Rosso (2016) | Study included CYP with a range of additional needs. Unclear how, specifically, autistic CYP were involved | |
| Schmidt et al. (2023) | Autistic adults | |
| Schmidt et al. (2024) | Autistic adults | |
| Sharpe et al. (2022) | Autistic CYP not included | |
| Sharpe et al. (2021) | Autistic CYP not included | |
| Ward et al. (2024) | Study was not conducted in a school setting. | |
| Zhu et al. (2021) | Study was not conducted in a school setting. | |
|  |  | |

**APPENDIX 3.** A summary of studies included in review

| **Reference** | **Country** | **Demographics** | **Approach & Method/s** | **Outcomes** |
| --- | --- | --- | --- | --- |
| **Benton and Johnson (2014)** | United Kingdom | Special Educational Provision - Autistic CYP aged 12-13 (*n* = 6). Male (*n* = 5), Female (*n* = 1) | Participatory Design – Creative idea generation (prototype development); language-based methods (Questionnaire) | Though autistic CYP enjoyed being part of the design process, difficulties arose when compromise was expected, resulting in the adult facilitator having to remediate conflicting ideas. While the study found that autistic CYP were able to contribute their ideas, prompting from adults was needed, and some needed help with their writing. It is unclear whether findings were shared with educators. |
| **Bossavit and Parsons (2017)** | United Kingdom | Special Educational Provision - male autistic CYP aged 15 (*n* = 2). | Participatory Design –creative idea generation (prototype development); language-based methods (guiding question; discussion with adults) | The authors noted that the two students gravitated towards their own preferences, with one working on design and the other on controls; autistic CYP also decided to work separately. A decrease in engagement was noted when the ICT teacher left the room, which led to off-task behaviour; in light of these issues, the authors commented that the role of the teacher is essential during design sessions. By designing the game, autistic CYP met curriculum requirements, yet the authors note that their approach could have been strengthened if autistic CYP were involved in early stages of the research. |
| **Bossavit and Parsons (2018)** | United Kingdom | Special Educational Provision - Autistic CYP aged 9 – 15 (*n* = 6). Male (*n* = 5), female (*n* = 1) | Participatory Design – Language-based methods (questionnaires); observation methods (video recording) | Autistic CYP completed a pre- and post-game questionnaire to test Geography knowledge. All sessions were video recorded and coded by the research team to provide insight into autistic CYP social interactions. Questionnaire data revealed that the game aided Geography learning, with an increase in correct answers reported after sessions. Video data indicated that the game improved social competencies. The authors did not comment whether findings were shared with educators. |
| **Constantin et al. (2017)** | United Kingdom | Special Educational Provision - Study 1: Autistic CYP aged 12 (*n* = 3). Male (*n* = 2), Female (*n* = 1). Study 2: Autistic CYP aged 11-13 (*n* = 4). Male (*n* = 3), Female (*n* = 1). Autistic CYP with ID (*n = 4).* Male (*n* = 2), Female (*n* = 2) | Participatory Design – Creative idea generation (card-sorting, prototype development, worksheets); language-based methods (mind-mapping questioning; discussion) | Autistic CYP were able to communicate their reward preferences verbally, through drawings, and through writing. Visual images were used to prompt ideas, and an adult scribed for one student. Though voices of autistic CYP were elicited, the researchers did not comment on whether the product of the design process was implemented in the school’s social stories programme. |
|  |  |  |  | *(Continued)* |
| **APPENDIX 3.** *(continued)* | |  |  |  |
| **Reference** | **Country** | **Demographics** | **Approach & Method/s** | **Outcomes** |
| **Danker et al. (2016)** | Australia | Mainstream – male autistic CYP aged 14-15 (*n* = 10). Two had mild ID and one ADHD. | Participatory Techniques – Visual methods (photovoice); language-based methods (photo-elicitation semi-structured interviews) | Autistic CYP were able to explain what they thought makes a good life in school using photographs that they had taken, with the authors commenting that photographs aided memory recall and lessened anxiety during interviews. Authors did not comment on its influence on school inclusive practices. |
| **Danker et al. (2019)** | Australia | Mainstream - autistic CYP aged 13-17 (*n* =16). Male (*n* = 15), female (*n* = 1). One had mild ID and one ADHD. | Participatory Research – Visual methods (photovoice); language-based methods (photo-elicitation semi-structured interviews) | Using photovoice, autistic CYP were able to conceptualise wellbeing, which in turn elucidated their barriers to learning. However, the authors commented that educators often hindered autistic CYP involvement and did not arrange follow up meetings for autistic CYP. Dissemination activities were ambiguously reported. |
| **Fage et al. (2019)** | France | Mainstream - autistic CYP aged 12-17 (*n* = 14). Gender not reported. | Participatory Design – Observation methods (teacher questionnaires, and parent interviews) | The study aimed to test the usability of an app designed to support emotional regulation. 14 autistic CYP were given the app to use; these results were compared with a control group of 15. Autistic CYP were placed into unfamiliar classes, where their emotional regulation was observed. While findings indicated that the app could support emotional regulation, the authors did not state whether the app was used by the school post-study. |
| **Fleming et al. (2023)** | United Kingdom | Special Educational Provision – Male autistic CYP aged 12-13 (*n* = 5). | Participatory Research – CYP as co-researchers; visual methods (photographs) | Autistic CYP were invited as co-researchers, and were able to evaluate an intervention using words, memes, pictures and symbols. Autistic CYP were empowered to own their work by being involved in the data analysis stage. While this provided a useful insight into autistic CYP perceptions, authors did not comment on its influence on school inclusive practices. |
|  |  |  |  | *(Continued)* |

| **APPENDIX 3.** *(continued)* | | | |  | |  | |
| --- | --- | --- | --- | --- | --- | --- | --- |
| **Reference** | **Country** | **Demographics** | **Approach & Method/s** | | **Outcomes** | |  |
| **Glass et al. (2024)** | United Kingdom | Mainstream – female autistic CYP aged 12-15 (*n* = 3). | | Participatory Research – visual methods (digital stories) | | Digital stories allowed autistic CYP to reflect on their experiences in school and had benefits beyond the project, with one student finding work experience with Autek following involvement in the project. Two of these stories were shared online, allowing educators to access them. | |
| **Goodall (2018)** | United Kingdom | Mainstream school and home school - autistic CYP aged 11-17 (*n* = 12). Male (*n* = 10), female (*n* = 2) | | Participatory Approach – Language-based methods (interviews); creative idea generation (bean and pots; diamond ranking; good teacher, bad teacher drawing; me at school drawing; school design drawing) | | Autistic CYP identified challenges surrounding mainstream school settings, such as bullying, class sizes, unpredictable environments, and a perceived lack of care from educators. Participants commented on how they wanted school to be, which included, more training for educators, increased awareness of autism, smaller class sizes, and clear schedules. The authors did not comment whether these findings were shared with educators. | |
| **Hoy et al. (2018)** | United Kingdom | Mainstream - autistic CYP aged 12-16 (*n* = 5). Male (*n* = 4), female (*n* = 1) | | Co-construction – Visual methods (photovoice); language-based methods (semi-structured interviews). | | Autistic CYP, accompanied by the researcher, took photographs around school. After this, interviews were conducted with autistic CYP to understand their reasons behind taking the photographs. These interviews were triangulated with teacher and parent interviews, which examined perceptions regarding school transitions. Findings indicated the benefit of visual supports, familiarisation, and communication, in transition planning. While findings gave school stakeholders a voice in decision-making, the authors did not comment on whether any changes happened as a result of the study. | |
|  |  |  | |  | | *(Continued)* | |

| **APPENDIX 3.** *(continued)* | | |  |  |
| --- | --- | --- | --- | --- |
| **Reference** | **Country** | **Demographics** | **Approach & Method/s** | **Outcomes** |
| **Hummerstone and Parsons (2022)** | United Kingdom | Mainstream - Autistic adolescents aged 12-13 (*n* = 6). Male (*n* = 5), female (*n* = 1) | Participatory Design - language-based methods (individual discussion; annotated worksheets) | Autistic CYP were optimistic about the motivation mixer but did not think that staff practice would change as a result; this was supported by interviews with staff who were concerned with resource and time constraints. |
| **Istuany and Wood (2020)** | Chile | Mainstream – male autistic CYP aged 10-11 (*n* = 4). One male autistic student aged 19 was also included. However, we focus on outcomes for those within our age range. | Participatory Approach - Creative idea generation through activity-based focus groups (tangible materials); language-based methods (questionnaires; individual interviews) | Autistic CYP were invited to comment on their inclusion in school through activity-based focus groups, individual interviews and questionnaires. While transcription of the data provided a useful insight into school inclusion, the authors did not comment on its influence on school inclusive practices. |
| **Lemaignan et al. (2024)** | United Kingdom | Special Educational Provision - Autistic CYP aged 12-16 (*n =* 30). Gender not reported. | Participatory Design – technology-based methods (observation); language-based methods (questionnaire) | Observation data collection was used to record autistic CYP interactions with the social robot, however this proved unreliable and was replaced with researcher observations. These observations indicated its popularity. Autistic CYP (*n =* 30) also completed questionnaires where they gave their opinions about the robot. Findings were shared with educators, and the value of the social robot was discussed. |
| **McAllister and Sloan (2016)** | United Kingdom | Mainstream - autistic CYP aged 11-18 (*n* = 17). Gender not reported. | Participatory Design – creative idea generation (design using jigsaw kit); language-based methods (question and answer) | Autistic CYP were given a jigsaw kit to design their ideal school layout. Finished designs were then photographed, and autistic CYP were asked to explain their design choice using question and answer. While the study gave autistic CYP opportunity to design their ideal school, the authors did not comment whether designs were shared with educators. |
|  |  |  |  | *(Continued)* |
| **APPENDIX 3.** *(continued)* | |  |  |  |
| **Reference** | **Country** | **Demographics** | **Approach & Method/s** | **Outcomes** |
| **Merter and Hasirci (2018)** | Turkey | Special Educational Provision - Autistic CYP aged 4-14 (*n* =8). Male (*n* = 6), female (*n* = 2). 1 male also had intellectual and physical disability. Another male had epilepsy, hyperactivity, and hyposensitivity. | Participatory Design – observation methods (direct observation; interviews and questionnaires with parents and teachers) | Initially, parents, teachers and a design team developed a trampoline intervention which would be safer for autistic CYP. Study was then tested with autistic CYP. CYP in the study were non-speaking and, as such, were involved through observation. While the study heightened understanding of autistic CYP needs and aided the development of a trampoline intervention the authors did not comment on whether the intervention continued post-study. |
| **Molloy and Farrell (2024)** | Ireland | Mainstream -Primary-aged autistic CYP (*n* =6); educators (*n* =6). Gender and age not reported. | Participatory Research – observation methods (interviews with teachers); students as co-researchers. | Autistic CYP trialled an intervention and were observed. Though teachers were optimistic about the use of the immersive technology intervention, the authors did not comment whether findings were shared with them, or whether the intervention continued post-study. |
| **Newbutt et al. (2023)** | United Kingdom | Special Educational Provision - Autistic CYP aged 11-14 (*n* = 10). Male (*n* = 8), female (*n* = 2) | Participatory Design – language-based methods (paper-based question and answers); observation (video recordings of CYP engaging with a social robot). | Sessions included autistic CYP interacting with a social robot following guiding questions from educators. In the final session, autistic CYP were given paper, pens, and pictures of the robot, and were asked to answer questions around the paper. Teaching assistants supported autistic CYP in responding to the questions, which included verbal responses, drawings, and writing. |
| **Newbutt & Bradley (2022)** | United Kingdom | Special Educational Provision - Autistic CYP aged 8-16 (*n* = 12). Male (*n* = 9), female (*n* = 3) | Participatory Research/ Co-participation – language-based methods (questionnaire; group discussion) | Autistic CYP were able to provide their views on VR. Autistic CYP initially had difficulties accessing the sense of presence inventory; as such, a questionnaire was co-produced with staff at the school *in lieu*. A lay summary of research outcomes were written by the school Trust and research team and published on the Trust’s website. As a result of the study, VR was incorporated into the school’s strategic plan and training. |
|  |  |  |  | *(Continued)* |

| **APPENDIX 3.** *(continued)* | |  |  |  |
| --- | --- | --- | --- | --- |
| **Reference** | **Country** | **Demographics** | **Approach & Method/s** | **Outcomes** |
| **Parsons (2015)** | United Kingdom | Mainstream - Autistic CYP aged 10-13 (*n* = 6); Gender not reported. | Participatory Design -Observation methods (video recordings) | Video recordings of autistic CYP engaging in Block Challenge indicated that the game could support social communication competencies. Teachers supported autistic CYP engagement through verbal cues, praise, and task re-direction. Unclear whether practical changes resulted from study. |
| **Parsons et al. (2021)** | United Kingdom | Special Educational Provision – Male non-speaking autistic CYP aged 4 (*n* = 5); school staff and parents. | Co-construction- visual methods (digital stories) | Video footage, detailing childrens’ daily activities, was recorded using wearcams attached to childrens’ tops, and allowed autistic CYP without words to communicate their preferences, e.g., preference for solitary play. Footage was edited by the research team. Videos helped teachers and parents to see how best to support autistic CYP. |
| **Suero** **Montero et al. (2023)** | Finland | Does not state whether participants were drawn from mainstream or specialist provisions - Autistic CYP/ CYP with autistic traits aged 10-12 (*n* = 8). Gender not reported. | Participatory Research – technology-based methods (eye tracking) language-based methods (visual stimulated account (VSA); questionnaires) | Participant gaze was measured through eye tracking devices. Video recordings were used to understand how autistic CYP interacted with their peers. The research provided understanding of the way autistic CYP socially interact. It is unclear whether findings were shared with educators. |
| **Tyrrell and Woods (2019)** | United Kingdom | Mainstream - autistic CYP aged 11-12 (*n* = 3). Male (*n* = 2), female (*n* = 1) | Action Research - creative idea generation (card sorting); language-based methods (interviews using visual prompts) | Autistic CYP were able to decide on their exam access arrangements. Autistic CYP and educators commented that the approach helped to match support to needs. Findings had relevancy to the school community beyond the project, with educators commenting that they planned to organise access arrangements in the same way in future and increase their flexibility in the type of support provided. However, it was unclear whether findings reached educators beyond those directly involved. |
| **Ward et al. (2022)** | United Kingdom | Special Educational Provision - Autistic CYP aged 13-16 (*n* = 14). Male (*n* = 12), female (*n* = 2) | Participatory Design –creative idea generation (prototype development); language-based methods (interviews; group discussion); visual methods (digital stories) | Autistic CYP were involved in designing a game which corresponded with their interests. After the design sessions, digital stories were used to portray autistic CYP’s experiences of the design process. It is unclear whether findings were shared with educators. |
|  |  |  |  | *(End of Table)* |

**APPENDIX 4.** Quality assessment of included studies. Yes (Y); No (N); Can’t tell (?); N/A (not marked) using MMAT (Hong et al., 2018)

|  | **Screening** | | **Qualitative studies** | | | | | | **Quantitative randomised controlled trials** | | | | | **Quantitative – nonrandomised** | | | | | **Quantitative descriptive** | | | | | | **Mixed methods** | | | | | | | |
| --- | --- | --- | --- | --- | --- | --- | --- | --- | --- | --- | --- | --- | --- | --- | --- | --- | --- | --- | --- | --- | --- | --- | --- | --- | --- | --- | --- | --- | --- | --- | --- | --- |
|  | **S1** | **S2** | **1.1** | **1.2** | **1.3** | **1.4** | **1.5** | **2.1** | | **2.2** | **2.3** | **2.4** | **2.5** | **3.1** | **3.2** | **3.3** | **3.4** | **3.5** | | **4.1** | **4.2** | **4.3** | **4.4** | **4.5** | **5.1** | | | **5.2** | | **5.3** | **5.4** | **5.5** |
| Benton and Johnson (2014) | Y | Y |  |  |  |  |  |  | |  |  |  |  |  |  |  |  |  | |  |  |  |  |  | Y | Y | | | Y | | N | Y |
| Bossavit and Parsons (2017) | Y | Y | Y | Y | Y | Y | ? |  | |  |  |  |  |  |  |  |  |  | |  |  |  |  |  |  |  | | |  | |  |  |
| Bossavit and Parsons (2018) | Y | Y |  |  |  |  |  |  | |  |  |  |  |  |  |  |  |  | |  |  |  |  |  | Y | Y | | | Y | | N | Y |
| Constantin et al. (2017) | Y | Y | Y | Y | Y | ? | Y |  | |  |  |  |  |  |  |  |  |  | |  |  |  |  |  |  |  | | |  | |  |  |
| Danker et al. (2016) | Y | Y |  |  |  |  |  |  | |  |  |  |  |  |  |  |  |  | |  |  |  |  |  |  |  | | |  | |  |  |
| Danker et al. (2019) | Y | Y | Y | Y | Y | ? | Y |  | |  |  |  |  |  |  |  |  |  | |  |  |  |  |  |  |  | | |  | |  |  |
| Fage et al. (2019) | Y | Y |  |  |  |  |  |  | |  |  |  |  | ? | Y | Y | N | Y | |  |  |  |  |  |  |  | | |  | |  |  |
| Fleming et al. (2023) | Y | Y | Y | Y | Y | ? | Y |  | |  |  |  |  |  |  |  |  |  | |  |  |  |  |  |  |  | | |  | |  |  |
| Frauenberger et al. (2019) | Y | Y | Y | Y | Y | ? | Y |  | |  |  |  |  |  |  |  |  |  | |  |  |  |  |  |  |  | | |  | |  |  |
| Glass et al. (2024) | Y | Y | Y | Y | ? | ? | Y |  | |  |  |  |  |  |  |  |  |  | |  |  |  |  |  |  |  | | |  | |  |  |
| Goodall (2018) | Y | Y | Y | Y | Y | ? | Y |  | |  |  |  |  |  |  |  |  |  | |  |  |  |  |  |  |  | | |  | |  |  |
| Hoy et al. (2018) | Y | Y | Y | Y | Y | ? | Y |  | |  |  |  |  |  |  |  |  |  | |  |  |  |  |  |  |  | | |  | |  |  |
| Hummerstone and Parsons (2022) | Y | Y | Y | Y | Y | ? | Y |  | |  |  |  |  |  |  |  |  |  | |  |  |  |  |  |  |  | | |  | |  |  |
| Istuany and Wood (2020) | Y | Y | Y | Y | Y | ? | Y |  | |  |  |  |  |  |  |  |  |  | |  |  |  |  |  |  |  | | |  | |  |  |
| Lemaignan et al. (2024) | Y | Y |  |  |  |  |  |  | |  |  |  |  |  |  |  |  |  | |  |  |  |  |  | Y | N | | | Y | | Y | Y |
| McAllister and Sloan (2016) | Y | Y | Y | Y | Y | ? | Y |  | |  |  |  |  |  |  |  |  |  | |  |  |  |  |  |  |  | | |  | |  |  |
| Merter and Hasirci (2018) | Y | Y | Y | Y | Y | ? | Y |  | |  |  |  |  |  |  |  |  |  | |  |  |  |  |  |  |  | | |  | |  |  |
| Molloy and Farrell (2024) | Y | Y | Y | ? | Y | Y | Y |  | |  |  |  |  |  |  |  |  |  | |  |  |  |  |  |  |  | | |  | |  |  |
| Newbutt et al. (2023) | Y | Y |  |  |  |  |  |  | |  |  |  |  |  |  |  |  |  | |  |  |  |  |  | Y | N | | | Y | | Y | Y |
| Newbutt et al. (2022) | Y | Y | Y | Y | Y | ? | Y |  | |  |  |  |  |  |  |  |  |  | |  |  |  |  |  |  |  | | |  | |  |  |
| Parsons (2015) | Y | Y | Y | Y | Y | ? | Y |  | |  |  |  |  |  |  |  |  |  | |  |  |  |  |  |  |  | | |  | |  |  |
| Parsons et al. (2021) | Y | Y | Y | Y | Y | ? | Y |  | |  |  |  |  |  |  |  |  |  | |  |  |  |  |  |  |  | | |  | |  |  |
| Suero Montero et al. (2023) | Y | Y |  |  |  |  |  |  | |  |  |  |  |  |  |  |  |  | |  |  |  |  |  | Y | Y | | | N | | N | Y |
| Tyrrell and Woods (2019) | Y | Y | Y | Y | Y | ? | Y |  | |  |  |  |  |  |  |  |  |  | |  |  |  |  |  |  |  | | |  | |  |  |
| Ward et al. (2022) | Y | Y | Y | Y | Y | ? | Y |  | |  |  |  |  |  |  |  |  |  | |  |  |  |  |  |  | |  | | |  |  |  |

*N.B*. “S1. Are there clear research questions? S2. Do the collected data allow to address the research questions? 1.1. Is the qualitative approach appropriate to answer the research question? 1.2. Are the qualitative data collection methods adequate to address the research question? 1.3. Are the findings adequately derived from the data? 1.4. Is the interpretation of results sufficiently substantiated by data? 1.5. Is there coherence between qualitative data sources, collection, analysis and interpretation? 2.1. Is randomization appropriately performed? 2.2. Are the groups comparable at baseline? 2.3. Are there complete outcome data? 2.4. Are outcome assessors blinded to the intervention provided? 2.5 Did the participants adhere to the assigned intervention? 3.1. Are the participants representative of the target population? 3.2. Are measurements appropriate regarding both the outcome and intervention (or exposure)? 3.3. Are there complete outcome data? 3.4. Are the confounders accounted for in the design and analysis? 3.5. During the study period, is the intervention administered (or exposure occurred) as intended? 4.1. Is the sampling strategy relevant to address the research question? 4.2. Is the sample representative of the target population? 4.3. Are the measurements appropriate? 4.4. Is the risk of nonresponse bias low? 4.5. Is the statistical analysis appropriate to answer the research question? 5.1. Is there an adequate rationale for using a mixed methods design to address the research question? 5.2. Are the different components of the study effectively integrated to answer the research question? 5.3. Are the outputs of the integration of qualitative and quantitative components adequately interpreted? 5.4. Are divergences and inconsistencies between quantitative and qualitative results adequately addressed? 5.5. Do the different components of the study adhere to the quality criteria of each tradition of the methods involved?” (Hong et al., 2018, p.2)
